# Cost-effectiveness of colorectal cancer screening in Ukraine

**DOI:** 10.1186/s12962-018-0104-0

**Published:** 2018-06-07

**Authors:** Nelya Melnitchouk, Djøra I. Soeteman, Jennifer S. Davids, Adam Fields, Joshua Cohen, Farzad Noubary, Andrey Lukashenko, Olena O. Kolesnik, Karen M. Freund

**Affiliations:** 1 Department of Surgery, Center for Surgery and Public Health, Brigham and Women’s Hospital/Harvard Medical School, 75 Francis St, Boston, MA 02115 USA; 2 Center for Health Decision Science, Harvard T.H. Chan School of Public Health, Boston, MA USA; 3 UMass Medical Center, Worcester, MA USA; 4 Tufts Clinical and Translational Science Institute, Boston, MA USA; 5 National Cancer Institute, Kiev, Ukraine; 6 Tufts Medical Center and Tufts University School of Medicine Boston, Boston, MA USA

## Abstract

**Background:**

Colorectal cancer is one of the most common cancers worldwide and is associated with high mortality when detected at a later stage. There is a paucity of studies from low and middle income countries to support the cost-effectiveness of colorectal cancer screening. We aim to analyze the cost-effectiveness of colorectal cancer screening compared to no screening in Ukraine, a lower-middle income country.

**Methods:**

We developed a deterministic Markov cohort model to assess the cost-effectiveness of three colorectal cancer screening strategies [fecal occult blood test (FOBT) every year, flexible sigmoidoscopy with FOBT every 5 years, and colonoscopy every 10 years] compared to no screening. We modeled outcomes in terms of cost per quality-adjusted life-years (QALYs) over a lifetime time horizon. We performed sensitivity analyses on treatment adherence, test characteristics and costs. Analyses were conducted from the perspective of the Ministry of Health of Ukraine.

**Results:**

The base-case lifetime cost-effectiveness analysis showed that all three screening strategies were cost saving compared to no screening, and among the three strategies, colonoscopy every 10 years was the dominant strategy compared to no screening with standard adherence to treatment. When decreased adherence to treatment was modeled, colonoscopy every 10 years was the most cost-effective strategy with an incremental cost-effectiveness ratio of $843 per QALY compared with no screening.

**Conclusion:**

Our findings indicate that colorectal cancer screening can save money and improve health compared to no screening in Ukraine. Colonoscopy every 10 years is superior to the other screening modalities evaluated in this study. This knowledge can be used to concentrate efforts on developing a national screening program in Ukraine.

## Background

The incidence of colorectal cancer (CRC) is increasing worldwide, making it the third most common cancer in men and the second most common in women [1, 2]. Mortality from CRC increases with advancing stage, with a significant drop in 5-year survival for stage IV disease. Mortality also correlates with the country’s gross domestic product (GDP), with higher mortality rates in countries with lower GDP [1, 3]. Similarly, the cost of colon cancer care depends on the stage at presentation, with costs including only surgery and surveillance for patients with early disease, but adds the cost of chemotherapy and/or radiation therapy for patients with more advanced disease [4]. The cost of chemotherapy can be prohibitive in low and middle income countries (LMIC) [5], while in these settings, the cost of surgery and surveillance can be manageable, given the reliance on local resources, including a physician workforce able to provide screening and treatment services, and inpatient hospital facilities available to provide care.

CRC is a perfect candidate for screening. Most CRCs develop from a precursor lesion, an adenomatous polyp, over a course of approximately 10 years [2]. Screening not only identifies CRC at an early stage but also can prevent the disease by identifying the precursor lesion that can be removed before it develops into cancer. Further supporting the potential benefits of CRC screening is the high incidence of CRC, its long preclinical and precancerous period, and the availability of treatment.

Multiple studies have demonstrated that CRC screening is cost effective in high-income countries with incremental cost-effectiveness ratios (ICERs) ranging from $611 to $103,000 per quality-adjusted life-year (QALY) [6–10]. In LMIC, however, there is a paucity of studies evaluating the cost-effectiveness of CRC screening. Ukraine is an example of a LMIC with reasonable local resources in terms of available physicians and infrastructure, but limited financial means. Currently, the country does not have an established CRC screening program and 35% of CRC patients die within a year of diagnosis, likely reflecting the late stage at diagnosis and lack of financial resources to make costly chemotherapy agents available to all patients [11]. In fact, 25% of patients with CRC in Ukraine do not receive any treatment [8].

The aim of this study was to assess the cost-effectiveness of various CRC screening strategies including no screening to inform the decision if implementing a screening program in Ukraine is cost-effective. We incorporated clinical and economic data from the published literature in a simulation model to compare the different CRC screening strategies over a lifetime time horizon in terms of costs per QALY.

## Methods

### Model overview

**Fig. 1:**
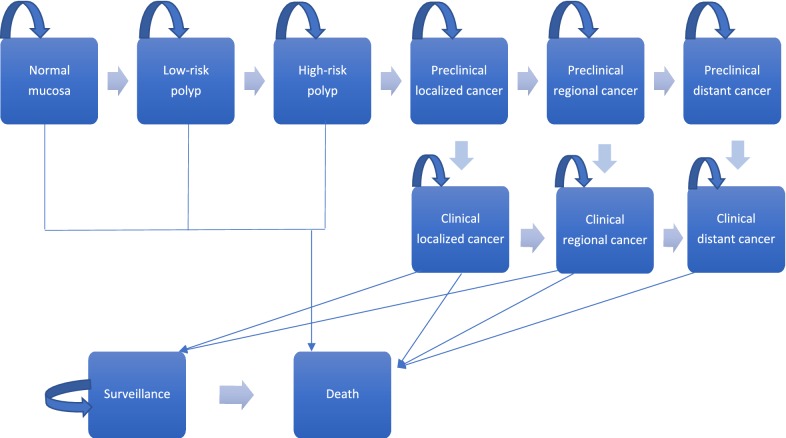
Natural history of colorectal cancer, Markov states

We modified a model structure of a previously-published Markov cohort model [7] to evaluate the cost-effectiveness of implementing a national screening protocol in Ukraine compared with the current situation of no screening (Fig. 1). We assumed that screening started for 50-year-old members of the population with an average cancer risk and that screening continued to the age of 75 years. We adopted the third party perspective and modeled clinical events and costs over a lifetime time horizon. The cycle length of the Markov model was 1-year. We implemented the model in TreeAge Pro (TreeAge Software Inc, Williamston, MA) and audited calculations via MS Excel. Table 1 lists the model parameters and key assumptions.

**Table 1:** Model parameters and assumptions

	Base case	Range (SD)	References
Natural history			
Prevalence of low risk polyp (based on age)	At age 50, 0.2	0.15–0.25	[15–17]
	At age 60, 0.4	0.35–0.45	
	At age 70, 0.5	0.45–0.55	
Prevalence of high risk polyp (based on age)	At age 50, 0.05	0.03–0.07	[15–17]
	At age 60, 0.09	0.07–0.12	
	At age 70, 0.16	0.14–0.18	
	At age 80, 0.21	0.20–0.22	
Prevalence of preclinical early CRC at age 50	0.0024	0.002–0.0026	[15–17]
Prevalence of preclinical regional CRC at age 50	0.0012	0.0008–0.0014	[15–17]
Prevalence of preclinical distant CRC at age 50	0.0004	0.0003–0.0005	[15–17]
Yearly transition probabilities			
Normal mucosa to low risk polyp (based on age)	At age 50, 0.00836	± 10%	[15–17]
	At age 55, 0.0099		
	At age 60, 0.01156		
	At age 65, 0.0133		
	At age 70, 0.01521		
Low risk polyp to high risk polyp	0.036	0.025–0.047	[15–17]
High risk polyp to preclinical local cancer	0.042	0.03–0.051	[15–17]
Preclinical local cancer to preclinical regional cancer	0.17	0.12–0.22	[15–17]
Preclinical regional to preclinical distal	0.10	0.05–0.15	[15–18]
Preclinical local to clinical local	0.17	0.12–0.23	[15–18]
Preclinical regional to clinical regional	0.21	0.16–0.26	[15–18]
Preclinical distant to clinical distal	1	N/A	[15–18]
Cancer mortality 5 year standard adherence			
Localized	0.1	N/A	[19]
Regional	0.35	N/A	[19]
Disseminated	0.92	N/A	[19]
Adherence with screening guidelines			
FOBT	0.75	0.4–0.8	[20–22]
Sigmoidoscopy with FOBT	0.75	0.4–0.8	[20–22]
Colonoscopy	0.8	0.4–0.8	[20–22]
Colonoscopy after positive screening test	0.84	0.4–0.9	[20–22]
Test characteristics			
FOBT sensitivity low risk polyp	0.03	0.01–0.1	[23, 24]
FOBT sensitivity high risk polyp	0.34	0.2–0.5	[23, 24]
FOBT sensitivity cancer	0.72	0.5–0.85	[23, 24]
FOBT specificity	0.91	0.7–0.96	[23, 24]
Colonoscopy/sigmoidoscopy sensitivity low risk polyp	0.92	0.75–0.95	[25–28]
Colonoscopy/sigmoidoscopy sensitivity high risk polyp	0.97	0.75–0.97	[25–28]
Colonoscopy/sigmoidoscopy sensitivity cancer	0.93	0.75–0.95	[25–28]
Colonoscopy/sigmoidoscopy specificity	1	N/A	[25–28]
Probability of negative sigmoidoscopy and proximal neoplasm	0.21	0.11–0.31	[25–28]
Complications			
Death from colonoscopic perforation	0.012	0.01–0.02	[30]
Perforation from diagnostic colonoscopy	0.0008	0.0006–0.005	[30]
QALY			
Local/regional cancer	0.7	0.52–0.9	[31]
Disseminated cancer	0.25	0.15–0.35	[31]
Costs (US $)			
Cost of colonoscopy	100	30–300	Expert opinion
Cost of FOBT	8	5–20	Manufacturer price
Cost of sigmoidoscopy	20	10–100	Expert opinion
Cost of treating colonoscopic perforation	500	200–1000	Expert opinion
Cost of local cancer treatment	500	200–1500	Expert opinion
Cost of regional cancer treatment	9000	500–15,000	[32]
Cost of disseminated cancer treatment	20,000	5000–25,000	[32]
Cost of surveillance	200	100–500	Expert opinion

*CRC* colorectal cancer, *FOBT* fecal occult blood test, *QALY* quality adjusted life years

Persons entered the model at the age of 50 and could reside in any of the following health states: normal mucosa, low risk polyp (< 1 cm), high-risk polyp (> 1 cm), preclinical CRC (localized, regional, and disseminated). They could transition between states in yearly time intervals, stay in their current state or develop clinical CRC (localized, regional, and disseminated). Persons could either die from cancer or from other causes or be in surveillance after treatment for colorectal cancer.

### Natural history

We used data from the published literature to obtain estimates of the prevalence of the polyps and age-specific transition probabilities [12–19] (Table 1). We adjusted the estimates of prevalence of the polyps to the incidence based on age of CRC in Ukraine. We used 2013 Ukrainian life tables from the World Health Organization for background mortality. We estimated stage-specific CRC mortality with treatment using US data from the Surveillance, Epidemiology and End Results (SEER) database, given that the data on CRC mortality in Ukraine doesn’t represent mortality with treatment since large proportion of patients to not receive cancer specific treatment [19]. We used the National Cancer Registry of Ukraine database to estimate current Ukrainian CRC incidence, mortality, and treatment adherence [8].

### Screening and treatment effectiveness

In this analysis we included three different screening strategies that would be reasonable to implement in a LMIC with an established medical infrastructure: (1) yearly FOBT, followed by confirmatory colonoscopy if positive; (2) flexible sigmoidoscopy every 5 years with FOBT yearly, also followed by colonoscopy if positive; (3) colonoscopy every 10 years [20–30]. Data on detection rate of these three screening scenarios, data on adherence with screening, screening complications rates, and utilities were obtained from screening studies conducted in Western countries and are displayed in Table 1 [17–22, 31]. We assumed no polyp removal during sigmoidoscopy, but instead that polyps are removed as part of a full colonoscopy procedure.

### Costs

We calculated costs from the third party payer perspective, in this case, the Ukraine Ministry of Health, and included direct treatment and screening costs. Indirect costs that are incurred by the patients such as transportation and lost productivity were not included. Screening costs were based on the manufacturer’s price for FOBT and on prices charged by private medical institutions in Ukraine (Table 1). We estimated treatment costs based on chemotherapy third party prices that the Ministry of Health of Ukraine pays for medications, as documented on the Ministry’s website [32]. We assumed doses appropriate for a 70 kg male and calculated costs for regional cancer [5-fluorouracil + Leukovorin + Oxaliplatin (FOLFOX for 12 cycles)] and disseminated cancer (FOLFOX + Bevacizumab for 12 cycles) regimens. We obtained surgical and radiation therapy costs from private clinics and modified them to reflect what would be reasonable in the public sector based on expert opinion from personnel at the National Cancer Institute of Ukraine (Personal communication with Dr. Kolesnik and Dr. Lukashenko). All costs were translated to 2012 US dollars.

### Cost-effectiveness analysis

We compared the four strategies by conducting a cost-effectiveness analysis. Strategies that were more costly and less effective than an alternative option were considered dominated and therefore excluded from the final cost-effectiveness calculations. For the remaining strategies, the incremental cost-effectiveness ratio (ICER) was calculated as the additional costs divided by the additional health benefit of the strategy compared with the next best non-dominated strategy. The most cost-effective strategy was then identified by comparing the ICERs against the willingness-to-pay (WTP) threshold for an additional QALY. We chose a WTP threshold of three times the per-capita gross domestic product (GDP), which is $11,700 US dollars per QALY (Ukraine’s annual per capita GDP is 3900 US dollars) [30]. The most cost-effective option was the strategy with the highest ICER below this WTP threshold. We discounted future costs and effectiveness at an annual rate of 3%, consistent with recommendations in the field of health economics [30].

### Sensitivity analyses

We conducted sensitivity analyses to determine the impact of different variables on the cost-effectiveness results. We tested the uncertain variables in the model in one-way sensitivity analyses. The variables that we varied were adherence with screening, test characteristics, rate of complications, and costs of screening strategies and treatment. The ranges were chosen based on existing literature for variation on natural history, test characteristics, and adherence parameters and based on expert opinion for cost parameters (Table 1). We conducted two and three-way sensitivity analyses on the assumptions that had the greatest effects on the cost-effectiveness results identified in the one-way sensitivity analyses.

## Results

### Model validation

**Fig. 2:**
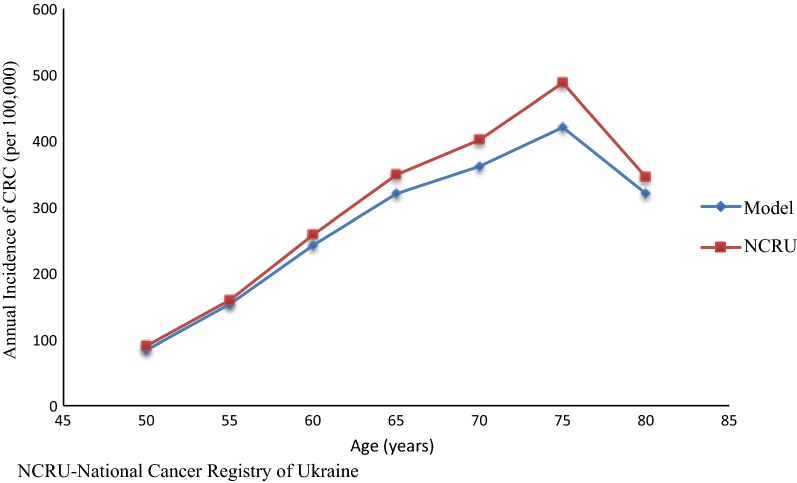
Model calibration: incidence based on age. *NCRU* National Cancer Registry of Ukraine

Figure 2 shows the annual incidence of CRC per 100,000 persons by age for the model predictions and data from the National Cancer Registry of Ukraine in 2013–2014. The figure illustrates that the observed incidences were very similar to what the model predicted.

### Cost-effectiveness analysis

**Fig. 3:**
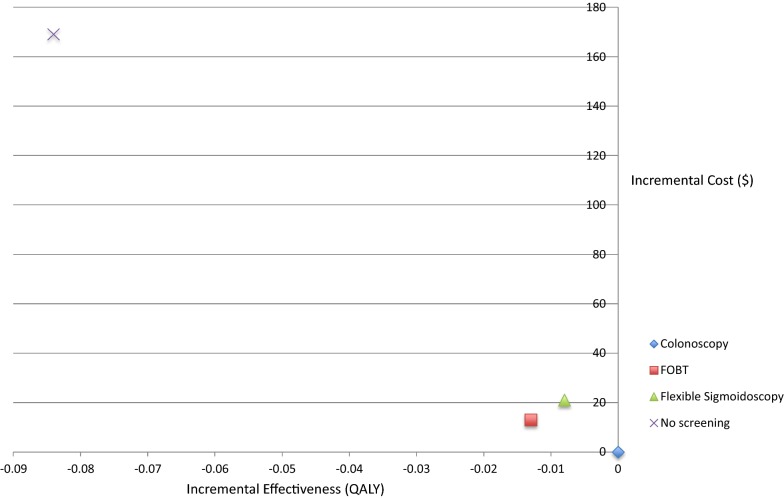
Cost-effectiveness plane. *FOBT* fecal occult blood test

The lifetime costs, effects, and cost-effectiveness of the different scenarios are presented in Table 2. All screening programs were less costly and more effective compared with the no screening program. FOBT yearly, sigmoidoscopy with FOBT every 5 years, and no screening were all more costly and less effective than colonoscopy every 10 years (Fig. 3).

**Table 2:** Costs, effects, and cost effectiveness of colorectal cancer screening programs over a lifetime horizon in the Ukraine

Strategy	Mean cost (2012, US$)	Mean effects (QALYs)	Incremental cost (2012 US$)	Incremental effects (QALYs)	ICER	Mortality decrease
Colonoscopy every 10 years	235	14.307	N/A	N/A	Dominant	73%
FOBT yearly	247	14.295	13	− 0.013	Dominated	61.6%
Sigmoidoscopy every 5 years with FOBT	256	14.300	21	− 0.008	Dominated	64%
No screening	404	14.218	169	− 0.084	Dominated	Reference

*US* United States, *QALY* quality adjusted life years, *ICER* incremental cost effectiveness ratios, *FOBT* fecal occult blood test

Mortality estimates (Table 2) demonstrate benefits with all three screening scenarios compared to no screening, with colonoscopy every 10 years producing the greatest benefits in terms of decreased mortality. Colonoscopy screening every 10 years decreased colon cancer mortality by 73% compared to no screening. FOBT decreased mortality by 61.6% and flexible sigmoidoscopy with FOBT decreased mortality by 64%.

### Sensitivity analyses

**Fig. 4:**
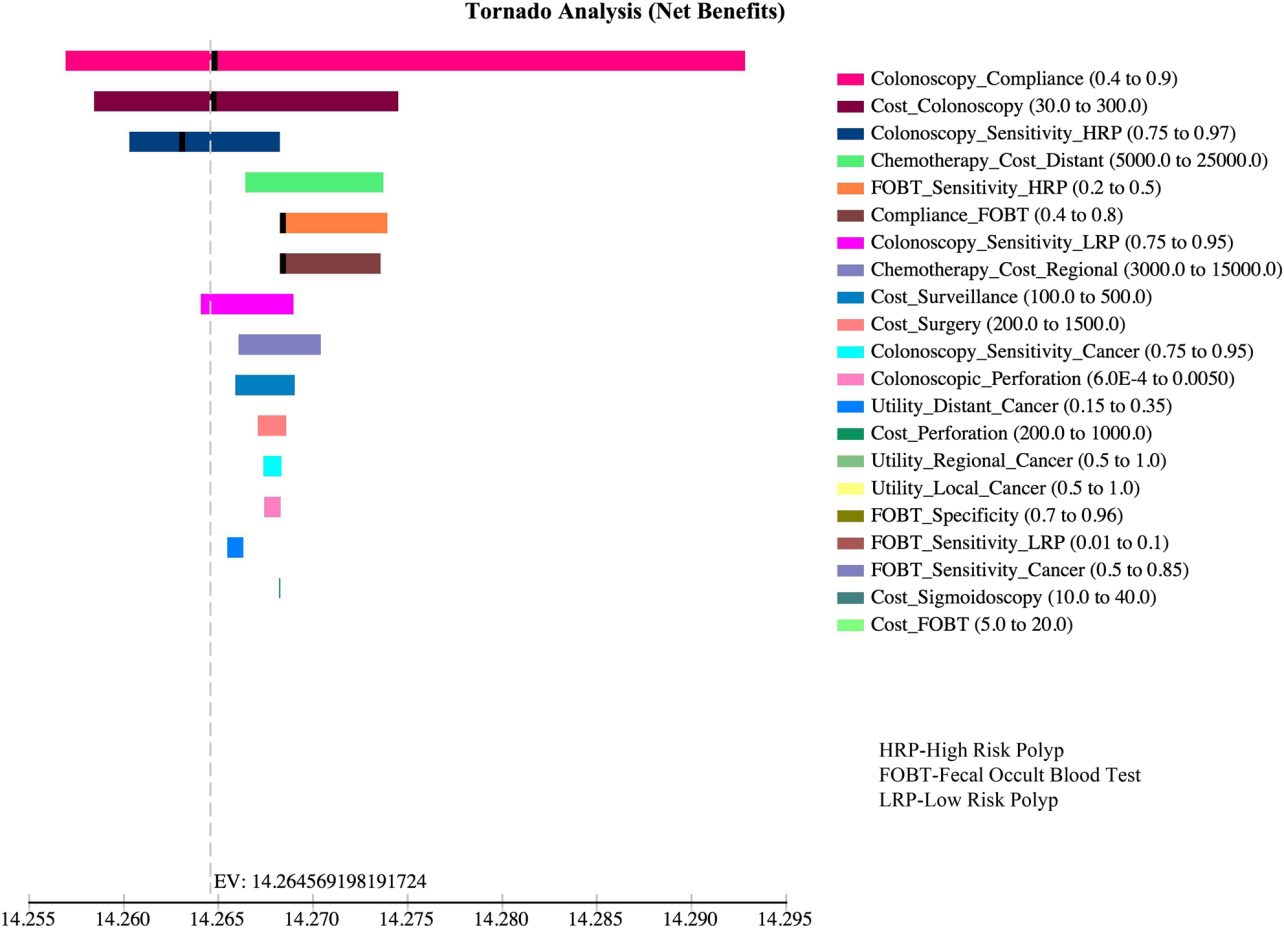
Tornado diagram

The results of the one-way sensitivity analyses show that the variable cost of colonoscopy and decreased compliance with colonoscopy have an impact on the most optimal screening scenario. The optimal screening scenario changes from colonoscopy every 10 years to sigmoidoscopy with FOBT when the costs of colonoscopy exceed the threshold value of $236 and the probability of compliance with colonoscopy falls below the threshold value of 0.66. The cost of cancer treatment or surveillance in the range tested did not impact the preferred strategy (Fig. 4).

In the scenario with decreased adherence to treatment, currently present in Ukraine, all the screening strategies were still cost effective, and the colonoscopy was still the most cost effective strategy with an incremental cost effectiveness ratio of $843.

## Discussion

Using a Markov cohort model, we compared the cost-effectiveness of three screening strategies compared to no screening starting in a 50 year-old population from the third party payer perspective. Our results indicate that the CRC screening strategies included in our analysis are cost saving in Ukraine compared to the current state of no national screening program. Colonoscopy every 10 years is the optimal screening program in terms of costs per QALY and decreased mortality. Results were sensitive to the cost of colonoscopy and adherence to screening. Even when we evaluated the scenario of lower adherence to cancer treatment, which is currently the situation in Ukraine, the results did not change with colonoscopy every 10 years as the preferred strategy with an ICER of $843, which is well below the WTP threshold for health of $11,700 per QALY.

There are multiple studies from the US and high-income European countries that demonstrated the cost effectiveness of CRC screening [8, 10, 33–37]. These interventions are usually reported to be cost-effective when their ICER is less than $50,000–$100,000 per QALY. Our findings are consistent with cost effectiveness studies done in the US and Europe, although most of those studies found colonoscopy or screening in general to be favorably cost-effective, whereas we found these strategies to both improve health and save money. This observation is not surprising given that the cost of colonoscopy is very low in Ukraine in contrast to the high cost of treatment of advanced stages of CRC [5].

Ukraine is similar to other countries in Central and Eastern Europe (CEE), which are characterized by significantly lower cancer survival then in Western European countries, and colorectal cancer is not an exception [38]. Less effective cancer control strategies, such as low screening rate, scarcity of screening programs, less treatment options available and cost of care among many others, have been implicated [39]. Focus on improved cancer control and prevention is of a paramount importance.

The reduction in CRC mortality with screening that we found is similar to the corresponding reductions reported by prior cost effectiveness studies, thus corroborating our model [7, 8, 40]. Given that a large proportion of the patients in Ukraine do not undergo the proper treatment for their CRC, the reduction in mortality and benefit in terms of costs and health benefits to society was even greater when low treatment adherence was considered.

Our analysis is not without limitations. The costs were calculated from the payer perspective. Taking the societal perspective, where the cost of lost productivity, cost of travel is included may be more appropriate but likely would not change the results given that colonoscopy is only performed every 10 years and other screening methods happen more frequently, with increased opportunity for societal costs. The model did not incorporate the potentially high cost of establishing a national screening program in Ukraine, including the costs of addressing adherence, public health campaigns to change attitudes, or other methods at the provider level to increase adherence.

To our knowledge this is the first cost-effectiveness analysis focusing on screening strategies for colorectal cancer in Ukraine and has the potential to contribute significantly to the knowledge base guiding rational decision making with respect to clinical practice and health care resource allocation [41]. If acted upon, the findings of our study may substantially improve CRC care in Ukraine and can be used to concentrate efforts on developing a national screening program. Additionally, this analysis can guide analyses in other LMIC countries with similar GDP and infrastructure.

## References

[1] Ferlay J (2010). Estimates of worldwide burden of cancer in 2008: GLOBOCAN 2008. Int J Cancer.

[2] Global Burden of Disease Cancer (2017). Global, regional, and national cancer incidence, mortality, years of life lost, years lived with disability, and disability-adjusted life-years for 32 cancer groups, 1990 to 2015: a systematic analysis for the global burden of disease study. JAMA Oncol..

[3] Jakovljevic MB, Vukovic M, Fontanesi J (2016). Life expectancy and health expenditure evolution in Eastern Europe-DiD and DEA analysis. Exp Rev Pharmacoecon Outcomes Res.

[4] Jakovljevic M (2015). Radiation therapy remains the key cost driver of oncology inpatient treatment. J Med Econ.

[5] Jakovljevic M (2014). Costs differences among monoclonal antibodies-based first-line oncology cancer protocols for breast cancer, colorectal carcinoma and non-Hodgkin’s lymphoma. J BUON.

[6] Frazier AL (2000). Cost-effectiveness of screening for colorectal cancer in the general population. JAMA.

[7] Telford JJ (2010). The cost-effectiveness of screening for colorectal cancer. CMAJ.

[8] Vijan S (2007). The cost-effectiveness of CT colonography in screening for colorectal neoplasia. Am J Gastroenterol.

[9] Lansdorp-Vogelaar I, Knudsen AB, Brenner H (2011). Cost-effectiveness of colorectal cancer screening. Epidemiol Rev.

[10] Zauber AG, et al. In cost-effectiveness of CT Colonography to screen for colorectal cancer. Rockville; 2009. 25834880

[11] Kovacevic A (2015). End-of-life costs of medical care for advanced stage cancer patients. Vojnosanit Pregl.

[12] Arminski TC, McLean DW (1964). Incidence and distribution of adenomatous polyps of the colon and rectum based on 1000 autopsy examinations. Dis Colon Rectum.

[13] Rickert RR (1979). Adenomatous lesions of the large bowel: an autopsy survey. Cancer.

[14] Vatn MH, Stalsberg H (1982). The prevalence of polyps of the large intestine in Oslo: an autopsy study. Cancer.

[15] Regula J (2006). Colonoscopy in colorectal-cancer screening for detection of advanced neoplasia. N Engl J Med.

[16] Strul H (2006). The prevalence rate and anatomic location of colorectal adenoma and cancer detected by colonoscopy in average-risk individuals aged 40–80 years. Am J Gastroenterol.

[17] Schoenfeld P (2005). Colonoscopic screening of average-risk women for colorectal neoplasia. N Engl J Med.

[18] Lieberman DA, Weiss DG, Veterans G, Affairs Cooperative Study (2001). One-time screening for colorectal cancer with combined fecal occult-blood testing and examination of the distal colon. N Engl J Med.

[19] Myers MH, Ries LA (1989). Cancer patient survival rates: SEER program results for 10 years of follow-up. CA Cancer J Clin.

[20] Selby JV (1992). A case-control study of screening sigmoidoscopy and mortality from colorectal cancer. N Engl J Med.

[21] Jorgensen OD, Kronborg O, Fenger C (2002). A randomised study of screening for colorectal cancer using faecal occult blood testing: results after 13 years and seven biennial screening rounds. Gut.

[22] Mandel JS (2000). The effect of fecal occult-blood screening on the incidence of colorectal cancer. N Engl J Med.

[23] Greenberg PD (2000). A prospective multicenter evaluation of new fecal occult blood tests in patients undergoing colonoscopy. Am J Gastroenterol.

[24] Imperiale TF (2004). Fecal DNA versus fecal occult blood for colorectal-cancer screening in an average-risk population. N Engl J Med.

[25] Collins JF (2005). Accuracy of screening for fecal occult blood on a single stool sample obtained by digital rectal examination: a comparison with recommended sampling practice. Ann Intern Med.

[26] Pickhardt PJ (2003). Computed tomographic virtual colonoscopy to screen for colorectal neoplasia in asymptomatic adults. N Engl J Med.

[27] Cotton PB (2004). Computed tomographic colonography (virtual colonoscopy): a multicenter comparison with standard colonoscopy for detection of colorectal neoplasia. JAMA.

[28] Irvine EJ (1988). Prospective comparison of double contrast barium enema plus flexible sigmoidoscopy v colonoscopy in rectal bleeding: barium enema v colonoscopy in rectal bleeding. Gut.

[29] Rex DK (1997). Colonoscopy and acute colonic pseudo-obstruction. Gastrointest Endosc Clin N Am.

[30] Levin TR (2006). Complications of colonoscopy in an integrated health care delivery system. Ann Intern Med.

[31] Ness RM (1999). Utility valuations for outcome states of colorectal cancer. Am J Gastroenterol.

[32] Ministry of Health, Ukraine. http://www.moz.gov.ua/ua/portal/register_medicines/. Accessed 15 June 2017.

[33] Zauber AG, et al. In evaluating test strategies for colorectal cancer screening-age to begin, age to stop, and timing of screening intervals: a decision analysis of colorectal cancer screening for the US preventive services task force from the cancer intervention and surveillance modeling network (CISNET). Rockville; 2009. 20722163

[34] Sonnenberg A, Delco F, Inadomi JM (2000). Cost-effectiveness of colonoscopy in screening for colorectal cancer. Ann Intern Med.

[35] O’Leary BA (2004). Cost-effectiveness of colorectal cancer screening: comparison of community-based flexible sigmoidoscopy with fecal occult blood testing and colonoscopy. J Gastroenterol Hepatol.

[36] Norum J (1998). Prevention of colorectal cancer: a cost-effectiveness approach to a screening model employing sigmoidoscopy. Ann Oncol.

[37] Ness RM (2000). Cost-utility of one-time colonoscopic screening for colorectal cancer at various ages. Am J Gastroenterol.

[38] De Angelis R (2014). Cancer survival in Europe 1999–2007 by country and age: results of EUROCARE-5-a population-based study. Lancet Oncol.

[39] Vrdoljak E (2016). Cancer control in central and Eastern Europe: current situation and recommendations for improvement. Oncologist.

[40] Leshno M, Halpern Z, Arber N (2003). Cost-effectiveness of colorectal cancer screening in the average risk population. Health Care Manag Sci.

[41] Jakovljevic MB (2013). Resource allocation strategies in Southeastern European health policy. Eur J Health Econ.

